# Article Watch

**DOI:** 10.7171/001c.162926

**Published:** 2026-06-11

**Authors:** Clive A Slaughter

**Affiliations:** 1 Biochemistry & Molecular Biology AU-UGA Medical Partnership

**Keywords:** Article Watch

## Abstract

This column highlights recently published articles that are of interest to the readership of this publication. We encourage ABRF members to forward information on articles they feel are important and useful to Clive Slaughter, AU-UGA Medical Partnership, 1425 Prince Avenue, Athens GA 30606. Tel; (706) 713-2216: Fax; (706) 713-2221: Email; cslaught@uga.edu or to any member of the editorial board. Article summaries reflect the reviewer’s opinions and not necessarily those of the Association.

## NUCLEIC ACIDS SQUENCING & GENOTYPING

**Arres J, Elavalli S, Behl S, Matias Sanchez D, Al Ali A, Saad A A Y A, Attia A, Minas C, Pariyachery S, Ahmed S, Aldhuhoori F, Thulasidharan N, Katagi G, Soliman O, Purohit S, Kusuma V, Cardenas R, Cardoso T, Paulin L F, Sanio P, Mafofo J, Wu H, Zvereff V, El-Khani A, Al Marzooqi F, Magalhães T R, Sedlazeck F J, Quilez J. Assessing the readiness of Oxford Nanopore sequencing for clinical genomics applications. *Genome Research* 36;460-471. DOI:** **10.1101/gr.280134.124**

Long-read sequencing (LRS) technology is supplied commercially by Pacific Biosciences (PacBio) (Menlo Park, CA, United States) and Oxford Nanopore Technologies (ONT) (Oxford, U.K.). These methodologies have significantly extended capabilities for the diagnosis and pathophysiologic study of genomic diseases. They enable accurate characterization of tandem repeat sequences, haplotype phases, and structural genomic variation in normal and malignant cells. They also enable identification of causative alleles within blocks of loci in linkage disequilibrium – a capability that standard short-read sequencing (SRS) lacks. Yet, SRS remains standard methodology for clinical genomic studies requiring whole-exome or whole-genome sequencing because of its accuracy in identifying single-nucleotide variants, for clinical testing must be validated under especially high standards of accuracy, reproducibility, and accessibility. Arres *et al.* here perform a benchmark study to establish the degree of readiness of ONT methodology for operation in this sphere. They address three key issues: firstly, whether the lower read accuracy of ONT impairs identification of variants; secondly, whether ONT is capable of processing large volumes of samples with fast turnaround times; and, thirdly, whether ONT is capable of identifying clinically important genetic variants. They present whole-genome sequence data for 17 well-characterized samples from the Coriell Institute for Medical Research. They utilize the most recent chemistries, R9 and R10, and analyze the data with a modified variant calling pipeline from ONT to detect single-nucleotide variants, indels, structural variants, copy number variants, and short tandem repeats. The results are compared with SRS results acquired with platforms from Illumina Inc (San Diego, CA, United States) and MGI Tech (Shenzhen, China). Among the findings, Arres *et al.* observe that ONT identifies single-nucleotide variants as accurately as SRS but still behaves substantially less well in detecting indels, although the newer R10 chemistry performs better than the older R9 in this respect. ONT performs as well as SRS in detecting all other disease-causing variants tested.

## METABOLOMICS

**Meikle T G, Wu J, Wang T, Dakic A, Yi C, Mellett N, van Buuren-Milne M, Beyene H B, Cinel M, Duong T, Gooley A, Kouremenos K, Whittle B, Shaw J E, Magliano D J, Yang J Y, Grieve S M, Vernon S T, Gray M P, Figtree G A, Huynh K, Giles C, Meikle P J. A Clinical lipidomics platform: development and validation of a high-throughput LC-MS assay for cardiovascular disease risk assessment. *Analytical Chemistry* 98;2026:9564-9576. DOI:** **10.1021/acs.analchem.5c04984**

Clinical decisions intended to reduce cardiovascular risk still rely heavily on routine serum measurements of total cholesterol, low-density lipoprotein (LDL), high density lipoprotein (HDL) and total triglyceride. Yet, methodology has long existed to quantify hundreds of serum lipid species, including many with known causative linkage to cardiovascular events, which are seldom measured. With a view to broaden the scope of data upon which clinical decisions are based, Meikle *et al.* design a platform for quantification of a much-expanded lipid panel with potential clinical impacts. Their clinical lipidomic platform comprises a combined liquid chromatography (LC), tandem mass spectrometry (MS/MS) assay that measures 248 human plasma lipid species from 37 lipid classes, plus 22 internal standards, with an effective cycle time of 6.5 min per sample (220 injections in a 24-hour period under routine operation). The analytes are presently chosen for their amenability to rapid, automated data processing. LC separation utilizes an Agilent 1290 Infinity II system with an Agilent 2.1 x 100 mm 95 Å reverse phase C18 column. MS utilizes an Agilent 649SC triple quadrupole instrument (Agilent Technologies, Santa Clara, CA, United States). A lipidomic risk score is formulated for a 10-year risk of cardiac events that incorporates lipidomic, clinical, and demographic data from the BioHEART-CT Discovery Cohort. This score is found to be highly correlated with the conventional Framingham Risk Score, but the new risk score outperforms the Framingham Risk Score in predicting the coronary artery calcium score that measures plaque burden, particularly in reassignment of individuals classified in the intermediate risk category by conventional measures, for whom the enhanced performance may yield the most benefit. The results encourage further development to integrate an expanded lipidomic platform into clinical practice.

**Zhang Y, Harms A, Jurado-Fasoli L, Ruiz J R, Drouin N, Hankemeier T. Direct infusion acoustic droplet ejection mass spectrometry: enabling high-throughput shotgun lipidomics. *Analytical Chemistry* 98;2026:9603-9616. DOI:** **10.1021/acs.analchem.5c05763**

Quantification of thousands of lipids in a biological sample is achievable by combining LC separation with MS/MS. However, throughput is limited by the duration of the LC separation. Throughput may be increased by direct injection into the mass spectrometer, but ion suppression and sample carryover during continuous infusion with contact-based injection systems arise as significant limitations. The instrument system described by Zhang *et al.* overcomes these limitations of direct sample introduction by employing direct infusion acoustic droplet ejection, in which focused acoustic energy ejects nanoliter-scale droplets of controlled size from samples reconstituted in octanol in microwell sample plates. The droplets directly enter an open port interface consisting of a transport capillary through which a carrier solvent is flowing at a rate of 350 µL/min. Carryover is minimized by the contactless design, and ion suppression is minimized by the large dilution of sample droplets in the carrier stream. This introduction system is incorporated commercially in the Echo MS system from SCIEX (Concord, Canada), coupled with a ZenoTOF 7600 mass spectrometer from SCIEX (Darmstadt, Germany), which supports multiple reaction monitoring. The system quantifies over 1000 lipid species across 14 lipid classes in less than 5 minutes per sample. It quantifies 731 lipid species in the Standard Reference Material 1950 human plasma sample from the National Institute of Standards and Technology. The chief limitation of the methodology presently remains its inability to resolve isomeric lipids.

## PROTEOMICS

**Guzman U H, Rykær M, Hendriks I A, Stewart H, Denisov E, Hagedorn B, Petzoldt J, Kreutzmann A, Mueller Y, Arrey T N, Colonius I, Østergaard O, Koenig C, Kraegenbring J, Fort K L, Couzijn E, Hauschild J-P, Hermanson D, Zabrouskov V, Hock C, Damoc E, Olsen J V. Higher-throughput proteome profiling enabled by parallelized pre-accumulation and optimized ion processing in the Orbitrap Astral Zoom mass spectrometer. *Molecular & Cellular Proteomics* 25;2026:101504. DOI:** **10.1016/j.mcpro.2025.101504**

Studies in proteomics that aim to identify new biomarkers of disease, or to determine new physiologic or pathophysiologic mechanisms, require not only deep proteomic coverage but also data acquisition from large numbers of individuals, samples, and measurement replicates. The scaling of analyses to meet these requirements demands minimization of MS measurement time without sacrificing depth of coverage or precision and accuracy of quantitative measurements. The necessary scaling involves shortening the chromatographic run times in LC-MS/MS analyses, maximizing mass spectral acquisition rates, and optimizing data analysis routines. Guzman *et al.* here report a benchmark study to assess the performance of the Orbitrap Astral Zoom MS system from Thermo Fisher Scientific (Waltham, MA, United States) in meeting these requirements. The Orbitrap Astral Zoom differs from its predecessor, the Orbitrap Astral, in improved preaccumulation of ions in the bent trap, a shorter duty cycle, and enhanced spectral processing for better deconvolution of overlapping features in MS/MS scans to increase the number of distinguishable product ions. The newer instrument increases MS/MS acquisition speed by 23% to 270 Hz. A protocol that uses data-independent acquisition achieves a throughput of 300 samples per day, utilizing a ∼216 second active chromatographic gradient. In human cell lines, it identifies ∼100,000 unique peptides representing proteins from ∼8,400 different genes.

Plubell D L, Remes P M, Wu C C, Jacob C C, Merrihew G E, Hsu C, Shulman N, MacLean B X, Heil L, Poston K L, Montine T J, MacCoss M J. Development of highly multiplex targeted proteomics assays in biofluids using a nominal mass ion trap mass spectrometer. *Molecular & Cellular Proteomics* 25;2026:101506. DOI: 10.1016/j.mcpro.2026.101506

Plubell *et al.* focus on the development of multiplexed mass spectrometric assays for proteins of biomedical interest to be used for transition from discovery experiments conducted with high resolution mass spectrometry to the simplified, robust, and validated assays for clinical decision-making with less advanced instrumentation. The instrument platform the authors chose for such translational work is the Stellar mass spectrometer from Thermo Fisher Scientific. The Stellar incorporates a quadrupole mass filter, followed by a multipole sector for routing and concentrating ions, followed by a dual pressure, radial ejection, linear ion trap. There is no Orbitrap. The data system dynamically accommodates shifts in chromatographic retention times during LC separation for scheduling the data-independent acquisition of product ion spectra from precursors on a preselected list. The quantification routine employs parallel reaction monitoring, with acquisition of product ion spectra at unit mass resolution. The instrument can quantify >2000 peptides in a single survey run. However, despite the need for maximizing multiplexing, the authors observe some trade-off between the number of precursor ions selected and the quantitative performance of the assays: fewer precursors provide better precision and lower limits of quantification. They develop an assay for 101 proteins in cerebrospinal fluid of interest in neurodegenerative disease.

**Yeung D, Lao Y, Spicer V, Paulo J, Zahedi R P, Krokhin O V. Selection of reversed-phase C18 separation media for bottom-up proteomics: multifactor comparison of fully porous sorbents including separation selectivity and carryover characteristics. *Journal of Proteome Research* 25;2026:2414-2423. DOI:** **10.1021/acs.jproteome.5c01175**

In recognition of the determinative role of the quality of chromatographic peptide separation in proteomics based on LC-MS/MS, a comparison of seven popular C18 reverse phase chromatographic stationary phases is reported in this study. Performance with respect to peptide identification output, peptide carryover, separation efficiency, and retentivity is documented under standard conditions of data-dependent mass spectral acquisition. Chromatography is performed in 0.1% formic acid, with gradient acetonitrile elution. The results are particularly interesting for the light they shed on the effects of stationary phase properties such as pore size and particle diameter. Pore size emerges as a major determinant of peptide retentivity. Reduction in pore size from 150 to 100 Å results in a diminution of peptide retention that may be attributed to reduced surface accessibility. Small peptides with a high charge density and increased ion pairing are relatively strongly affected. Numbers of identifications are sensitive to separation efficiency and retentivity, generally favoring smaller particle size and higher retentivity. The results are also of interest for retention time modeling: they indicate how retention times predicted by a model are likely to differ upon exchanging stationary phases.

**Starovoit M R, Jadeja S, Kupčík R, Vatić S, Rasl J, Demir D, Novák P, Braswell C, Orsburn B C, Lenčo J. Propionic acid outperforms formic and acetic acid in MS sensitivity for high-flow reversed-phase LC-MS bottom-up proteomics. *Analytical Chemistry* 98;2026:10572-10583. DOI:** **10.1021/acs.analchem.5c07595**

In a salutary lesson about uncritically following tradition, Starovoit *et al.* show that replacing the conventional mobile phase, 0.1% formic acid, with 0.5% propionic acid for on-line reverse phase LC-MS yields an average increase of 39% in peptide identifications due to enhanced ionization efficiency; and 0.5% propionic acid even yields 12% more identifications than the recently championed 0.5% acetic acid! The improvement is ascribed to reduced ionic strength, reduced surface tension, and reduced volatility. Sensitivity enhancement is seen in the microflow and nanoflow regimes in addition to conventional flow. Chromatographic performance is comparable, although peptide retention is somewhat decreased with 0.5% propionic acid, producing a slight decrement with hydrophilic peptides. Background noise is comparable, as is instrument corrosion.

## IMAGING

**Gasparin F, Prebeck A, Soldà A, Uluç N, Glasl S, Berger C, Pleitez M A, Ntziachristos V. Differentiation of sphingomyelin and cholesterol by hyperspectral mid-infrared detection of single-bond vibrational modes in the fingerprint region. *Nature Methods* 23;2026:815-822. DOI:** **10.1038/s41592-026-03025-w**

Optoacoustic imaging is an optical imaging modality in which transient light absorbed by cells or tissues causes small temperature changes in the presence of absorbing molecules, which set up local thermoelastic cycles of local expansion and contraction, establishing ultrasonic waves detectable with high spatial resolution. The selectivity imposed by the wavelengths of excitatory light absorbed by different molecules in tissues provide information about the chemical composition of the tissue. The term hyperspectral imaging refers to collection of images across a range of excitatory wavelengths for this purpose. The depth of imaging is determined by the penetration of light, which depends on the tissue and the optical wavelength. Microscopic application of optoacoustic imaging is well documented. Because the methodology is noninvasive, is nondestructive, and does not rely on the introduction of substances for chemical labeling, the technique has also found application in clinical imaging, notably in the field of oncology, but further development in its capabilities for discrimination of lipid species and metabolites are expected to expand its potential clinical utility. The present study illustrates the chemical discrimination that the methodology can provide. The authors first conduct a microscopic study of synthetic, unilamellar vesicles. They show that, with irradiation in the mid-infrared range, discrimination of cholesterol, sphingomyelin, and unsaturated phosphatidylcholine is possible with hyperspectral imaging. They then demonstrate deconvolution of spectral images for discrimination of these lipids in cultured cells. The next step will be to test the methodology for imaging of tissues at depth.

**Wang F, Sun C, Wu T W, Fu Y, Fan Y, Zhao S, Huang K, Pan Z, Lu Y, Han J R, Jia S, Zeng L, Zhang S, Chen T, An S, Meng S S, Guo X, Li W, Lian H, Sun X, Hu J, Yang C, Feng S, Li P, Du L, Liu X, Piatkevich K D, Zou Y. iPEX enables micrometre-resolution deep spatial proteomics via tissue expansion. *Nature* 649;2026:505-514. DOI:** **10.1038/s41586-025-09734-0**

Wang *et al.* couple mass spectral tissue imaging by matrix-assisted laser desorption/ionization mass spectrometry (MALDI) with hydrogel-mediated tissue expansion to improve spatial resolution. Proteins in chemically or cryogenically fixed tissue sections are anchored to a hydrogel mesh via *N*-succinimidyl acrylate. They are then reduced and alkylated. Next, the hydrogel is expanded by a factor of 3–7x with ammonium bicarbonate. Expanded sections are transferred to an indium tin oxide-coated slide, and proteins are digested by a sprayed protease. Finally, MALDI matrix (α-cyano-4-hydroxycinnamic acid) is applied to the desiccated slide by spraying or sublimation. MALDI imaging using this protocol yields an effective pixel size of 1–5 µm. The hydrogel serves to impede diffusional spread of peptides, favoring high spatial resolution. The authors further observe a very high signal strength with this protocol and associated high rates of protein identification. To explain this finding, they speculate that hydrogel expansion effectively dilutes substances competing for ionization with peptide analytes. The results represent a significant enhancement in the capabilities of MALDI mass spectral imaging.

## MACROMOLECULAR SYNTHESIS & SYNTHETIC BIOLOGY

**Pelea O, Tálas A, Carrera J F, Mathis N, van de Venn L, Yeh C D, Kulcsár P I, Marquart K F, Weber Y, Gerecke S E, Harvey-Seutcheu I F, Mailänder D, Pfleiderer M M, Chanez C, Corn J E, Schwank G, Jinek M. Programmable genome editing in human cells using RNA-guided bridge recombinases. *Science* 391;2026:eadz1884. DOI:** **10.1126/science.adz1884**

**Perry N T, Bartie L J, Katrekar D, Gonzalez G A, Durrant M G, Pai J J, Fanton A, Martins J Q, Hiraizumi M, Ricci-Tam C, Nishimasu H, Konermann S, Hsu P D. Megabase-scale human genome rearrangement with programmable bridge recombinases. *Science* 391;2026:eadz0276. DOI:** **10.1126/science.adz0276**

CRISPR-Cas nucleases, base editors, and prime editors are efficient at producing small-scale edits, but they are limited in their ability to insert large DNA fragments in a site-directed manner. For the latter purpose, new techniques that might offer a performance superior to the present capabilities of homology-directed repair, integrases, and transposons are actively being sought. A family of bacterial bridge RNA-guided recombinases exists that shows promise. The bridge RNA these enzymes use to determine specificity is a bipartite structure that has a target-binding loop and a donor-binding loop, each of which recognizes a 14-nucleotide sequence. The prototype enzyme in the family can be programmed to mediate deletions, inversions, and insertions in bacteria, but two groups now discover a new member of the bridge recombinase family from *Citrobacter rodentium*, named ISCro4, that works in human cultured cells. Pelea *et al.* enhance the activity of ISCro4 by splitting the bridge RNA into independent target-binding and donor-binding loops. They achieve recombination frequencies of >10% for deletions and inversions in genome-targeted reporter cell lines and achieve >6% frequency for insertion of exogenous multikilobase DNA fragments at endogenous positions. Perry *et al.* enhance the activity of ISCro4 by engineering the sequence of its bridge RNA. They investigate the determinants of target specificity, achieving up to 82% specificity. They also optimize the amino acid sequence of the recombinase enzyme. They observe 20% insertion efficiency in human cells. They also perform intrachromosomal inversion and excision of up to 0.93 megabases of DNA. Both groups go on to perform excision of disease-relevant sequences. Specificity is somewhat limited by the short (14-nucleotide) recognition sites, but future mutational enhancements may be expected in both activity and specificity. The present preliminary results foreshadow a new era of programmable genome design and therapeutic intervention.

Rackl J W, Boll L B, Wennemers H. Organocatalyst-controlled stereoselective head-to-tail macrocyclizations. *Science* 391;2026:931-936. DOI: 10.1126/science.aec8992

Macrocyclic compounds, which consist of rings with 12 or more atoms, are extensively used as pharmaceuticals. They are generally synthesized by cyclization of a flexible linear precursor. This process is frequently complicated and is rendered even more so when cyclization creates stereochemical centers whose chirality must be controlled. The present authors devise a cyclization method in which a bifunctional tripeptide is used as a catalyst to tether the two ends of the linear precursor together. The tripeptide is a chiral molecule that acts as a template to impose chirality on the macrocycle that’s formed. One functional group in the tripeptide is a secondary amine that forms a transient covalent enamine bond with an aldehyde at one end of the linear precursor. The other functional group in the tripeptide is a carboxyl group that forms a hydrogen bond with a vinyl ketoester or ketamide at the other end of the precursor. The tripeptide tethers the two ends in a conformation that favors stereoselectivity of the resulting bond between them. The authors synthesize 12- and 18-membered macrocyclic lactones and lactams in this way, and they demonstrate >99% enantiomeric excess and diastereomeric ratios of >20:1. The approach is validated by synthesizing the core of the natural product robotnikinin, which selectively binds to the Sonic Hedgehog protein.

## DESIGN & DEVELOPMENT OF THERAPEUTICS

**Zhang K, Tao H, Zhu D, Yue Z, Hu S, Wu Y, Yan N, Hu Y, Liu S, Liu M, Vahl T P, Ranard L S, Cheng X, Romanov A, Liu J, Zhang S W, Li Y, Lu C, Shen M, Lewis A, Huang K, Cheng K. Single intramuscular injection of self-amplifying RNA of *Nppa* to treat myocardial infarction. *Science* 391;2026:edau9394. DOI:** **10.1126/science.adu9394**

Zhang *et al.* explore methodology by which RNA administered therapeutically might express larger quantities of encoded protein than those achieved by conventional delivery of messenger RNA. Higher expression levels are important when successful treatment of a clinical condition requires larger quantities of the protein to mediate a targeted physiologic process. For such a purpose, the authors use self-amplifying RNA – double-stranded RNA that encodes not only the payload protein but also an RNA-dependent RNA replicase to amplify the quantity of RNA driving payload protein synthesis within cells. The authors deliver the RNA in lipid nanoparticles with a composition previously used in a COVID-19 vaccine. In proof of principal studies, they inject the preparation into neonatal mice and pigs via an intramuscular route. They apply this methodology to the preservation of cardiac function following myocardial infarction by expressing pro-atrial natriuretic peptide (pro-ANP). Most of the pro-ANP expression appears to occur close to the injection site. A single injection produces pro-ANP secretion for periods in excess of four weeks. The cardiac protease corin processes the pro-hormone to its active form. The ANP promotes cardiomyocyte proliferation and suppresses profibrotic fibroblast proliferation. The effect is to improve ventricular ejection fraction and reduce infarct size and fibrosis. The major difficulty with an approach utilizing self-amplifying RNA derives from the powerful stimulation to interferon γ release that double-stranded RNA causes. The authors seek to reduce inflammation at the site of injection by incorporating modified nucleotides into the RNA, which promotes protein expression and reduces expression of inflammatory markers.

